# The effective combination therapies with irinotecan for colorectal cancer

**DOI:** 10.3389/fphar.2024.1356708

**Published:** 2024-02-05

**Authors:** Yun Chai, Jing-Li Liu, Shuo Zhang, Na Li, Ding-Qiao Xu, Wen-Juan Liu, Rui-Jia Fu, Yu-Ping Tang

**Affiliations:** ^1^ Key Laboratory of Shaanxi Administration of Traditional Chinese Medicine for TCM Compatibility, and Shaanxi Key Laboratory of Chinese Medicine Fundamentals and New Drugs Research, Shaanxi University of Chinese Medicine, Xianyang, Shaanxi, China; ^2^ State Key Laboratory of Quality Research in Chinese Medicine, Macau University of Science and Technology, Taipa, Macao SAR, China

**Keywords:** colorectal cancer, irinotecan, cancer therapy, drug combinations, targeted drug, herbal medicine

## Abstract

Colorectal cancer is the third most common type of cancer worldwide and has become one of the major human disease burdens. In clinical practice, the treatment of colorectal cancer has been closely related to the use of irinotecan. Irinotecan combines with many other anticancer drugs and has a broader range of drug combinations. Combination therapy is one of the most important means of improving anti-tumor efficacy and overcoming drug resistance. Reasonable combination therapy can lead to better patient treatment options, and inappropriate combination therapy will increase patient risk. For the colorectal therapeutic field, the significance of combination therapy is to improve the efficacy, reduce the adverse effects, and improve the ease of treatment. Therefore, we explored the clinical advantages of its combination therapy based on mechanism or metabolism and reviewed the rationale basis and its limitations in conducting exploratory clinical trials on irinotecan combination therapy, including the results of clinical trials on the combination potentiation of cytotoxic drugs, targeted agents, and herbal medicine. We hope that these can evoke more efforts to conduct irinotecan in the laboratory for further studies and evaluations, as well as the possibility of more in-depth development in future clinical trials.

## Graphical Abstract

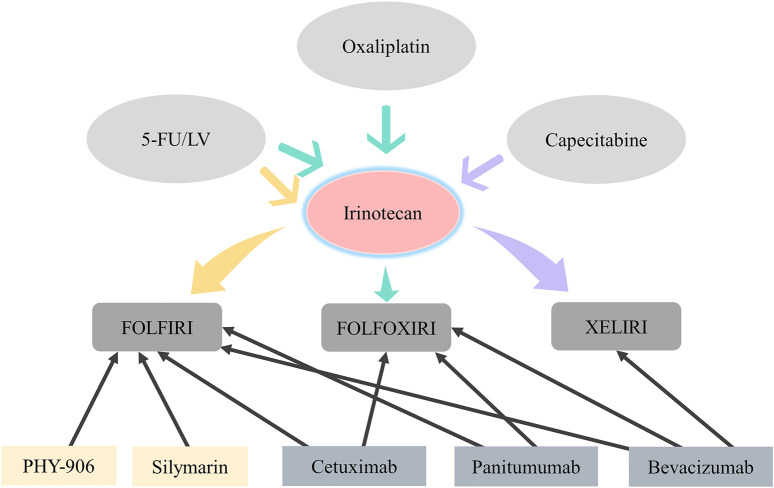

## 1 Introduction

In terms of incidence, colorectal cancer (CRC) is the second most frequent cause of cancer-related fatalities (Sung et al., 2021). Because of its high incidence and mortality worldwide, CRC has emerged as a global public health issue (Kumar et al., 2021). With the prevalence of CRC getting younger, it is expected that the economic burden will further increase, bringing great challenges to global public health (Siegel et al., 2023).

Currently, the treatment of CRC mainly includes chemotherapy, targeted therapy, radiation therapy, immunotherapy and palliative care. Chemotherapy is one of the vital means for the treatment of CRC. Chemotherapy can cooperate with other treatment methods and improve the effect of comprehensive treatment. Irinotecan is a critical component of the therapy of CRC and is typically used with other drugs to ease cancer-related symptoms and increase patients’ survival times. Irinotecan is a comprehensive anticancer therapy when combined with other drugs, such as oxaliplatin, capecitabine, 5-fluorouracil (5-FU) and leucovorin and other drugs composed of traditional chemotherapy. Molecularly targeted agents are included in the standard treatment of traditional chemotherapy, usually choosing between anti-epidermal growth factor receptor (EGFR) (cetuximab, panitumumab) and anti-vascular endothelial growth factor (VEGF) (bevacizumab) monoclonal antibodies. Although irinotecan-based combination chemotherapy improves the treatment and the survival in CRC patients, adverse events such as delayed diarrhea and neutropenia caused by irinotecan greatly limit clinical application. Therefore, it is particularly important to find treatment options with better specificity for CRC (Islam et al., 2022). At present, many studies have demonstrated that herbs can serve as an adjunctive in chemotherapy regimens related to irinotecan (Li et al., 2020; Sun et al., 2021). Herbs have the advantages of low toxicity, safety, effectiveness, and multi-targets (Qiu et al., 2018). Most importantly, herbs may achieve favorable therapeutic outcomes by alleviating the serious side effects caused by irinotecan, improving the quality of life (QoL) of patients and increasing the effectiveness of chemotherapy (Yue et al., 2021; Wang et al., 2021; Chen et al., 2022). However, their possible toxicity and side effects need to be evaluated over time. It is without a doubt that irinotecan is the main anticancer drug, no matter what type of drug it is combined with.

### 1.1 Metabolism of irinotecan

**FIGURE 1:**
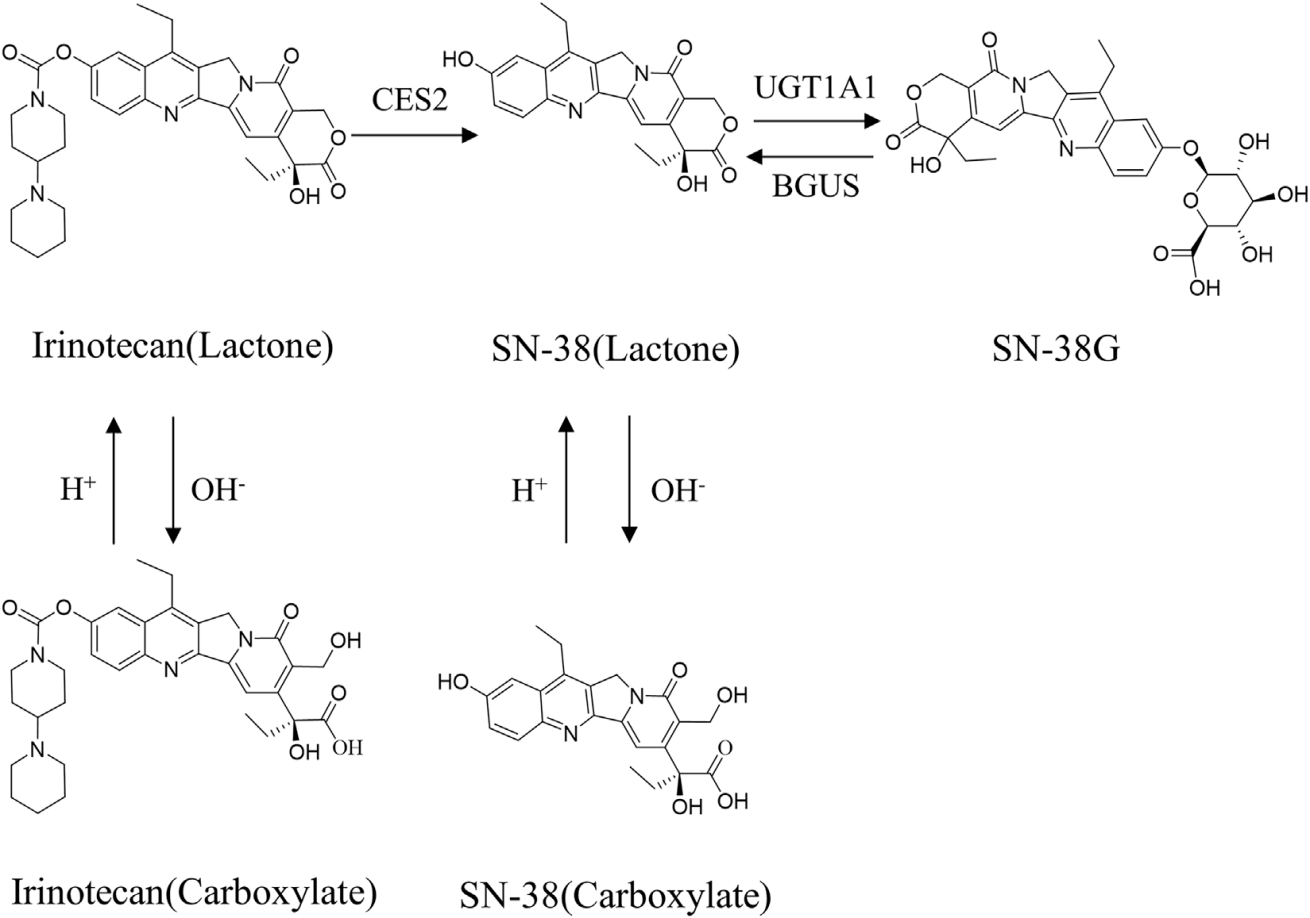
Schematic diagram of the *in vivo* transformation of irinotecan.

**FIGURE 2:**
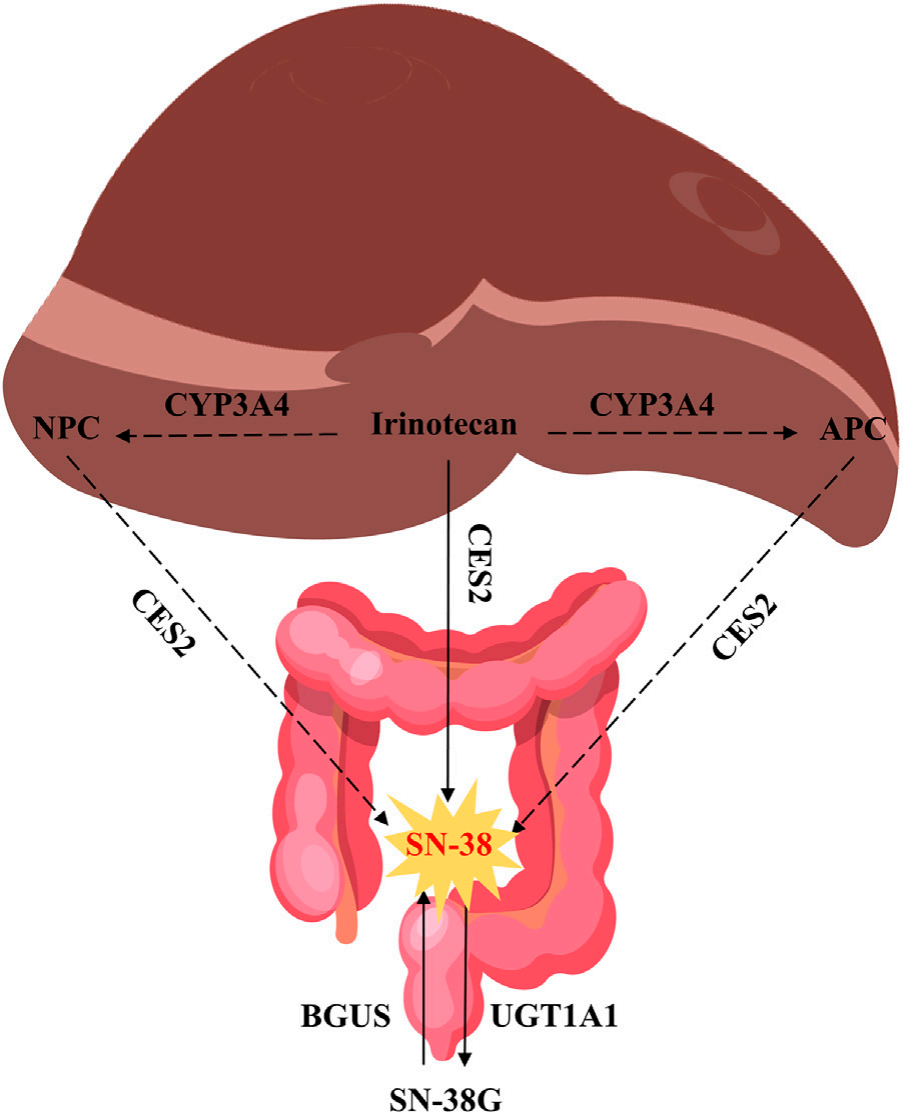
Metabolism of irinotecan. Source of liver and intestine illustrations: https://scidraw.io/.

Irinotecan, like other camptothecin derivatives, presents dynamic equilibrium in aqueous solution in two forms: one in lactone form and the other in carboxyl form, and the equilibrium constant of this reaction is pH dependent (Figure 1). In an acidic environment, the preference is for the lactone form. It is generally believed that lactone has anti-tumor activity, while carboxylate has no inhibitory effect on tumors (Thomas and Pommier, 2019). The distribution of irinotecan *in vivo* is thought to be mediated by various enzyme systems. In phase Ⅰ metabolism, irinotecan passes through the peripheral bloodstream to the liver, where it is metabolized *in vivo* to the active metabolite 7-ethyl-10-hydroxy-camptothecin (SN-38) by the catalytic action of carboxylesterase2 (CES2) (Mathijssen et al., 2001). Irinotecan is oxidized by the cytochrome P450 isoenzyme 3A4 (CYP3A4), resulting in the production of relatively inactive metabolites 7-ethyl-10-[4-N-(5-aminopentanoic acid)-1-piperidino] carbonyloxy camptothecin (APC) and a smaller amount of metabolites 7-ethyl-10-(4-amino-1-piperidino) carbonyloxy camptothecin (NPC) (Alimonti et al., 2004). NPC and APC can be further converted to SN-38 by CES2. The binding reaction of phase Ⅱ is mainly the glucuronidation process of active metabolite SN-38. SN-38 can be rapidly metabolized to inactive SN-38 glucuronide (SN-38G) by liver uridine diphosphate-glucuronosyltransferase 1A1 (UGT1A1) (Yue et al., 2021). The elimination of irinotecan is mainly liver metabolism and bile secretion. After SN-38G is secreted into the intestinal tract through bile, it is transformed into SN-38 under the action of β-glucuronidase (BGUS) produced by the intestinal tract (Figure 2), which is reabsorbed into the blood, causing dose-limited diarrhea (de Jong et al., 2006).

### 1.2 Anti-tumor mechanism of irinotecan

CRC is a disease that occurs in the colon or rectum and is caused by the abnormal proliferation of colonic glandular epithelial cells. The progression of CRC is a dynamic process based on the depth of tumor infiltration, the degree of lymph node invasion, and the presence of metastases in other organs (Hossain et al., 2022). It can be divided into five disease stages: benign polyp (stage 0), invades the muscularis propria (stage Ⅰ), invades tissue in the serosa (stage Ⅱ), invades visceral peritoneum (stage Ⅲ), metastasis to other organs (stage Ⅳ) (Mahmod et al., 2022). The liver is the most common site of metastasis, followed by the lung and bone. A key factor in the treatment of patients with mCRC is to maximize the likelihood of resection. Irinotecan-based chemotherapy can shrink tumors to the point of complete resection. Triple therapy with irinotecan (FOLFOXIRI) is more toxic compared to doublet therapy (FOLFIRI), but has advantages in terms of resectability of liver metastases, and XELIRI can be used as an alternative to FOLFIRI (Sobrero and Bennicelli, 2010).

**FIGURE 3:**
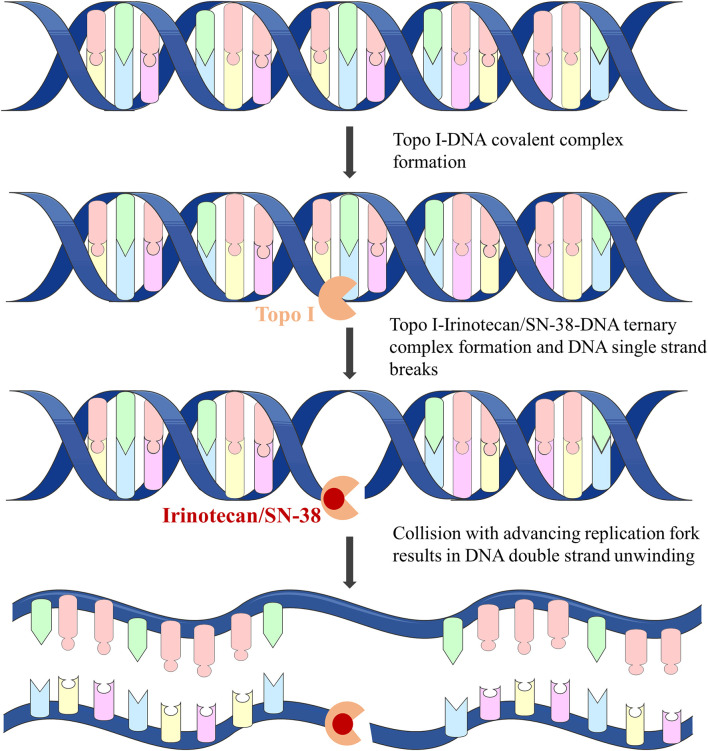
Anti-tumor mechanism of irinotecan.

Irinotecan is the most widely studied first- and second-line anti-CRC drug. It is both a derivative of camptothecin and a prodrug of SN-38. Irinotecan is a Topoisomerase Ⅰ (Topo Ⅰ) inhibitor and does not interact directly with DNA. Irinotecan and its active metabolite SN-38 combine with the Topo Ⅰ-DNA complex to form the Topo Ⅰ-Irinotecan/SN-38-DNA ternary complex by reversibly breaking DNA single strands (Xu and Villalona-Calero, 2002). When the ternary complex collides with the progressive replication fork, it will form double-stranded DNA unwinding (Figure 3), resulting in irreversible stagnation of the replication fork and cell death, and play a highly effective anti-tumor effect by interfering with the process of DNA replication in tumor cells (Bailly, 2019; Kciuk et al., 2020).

## 2 Combination therapies based on irinotecan

### 2.1 FOLFIRI

**FIGURE 4:**
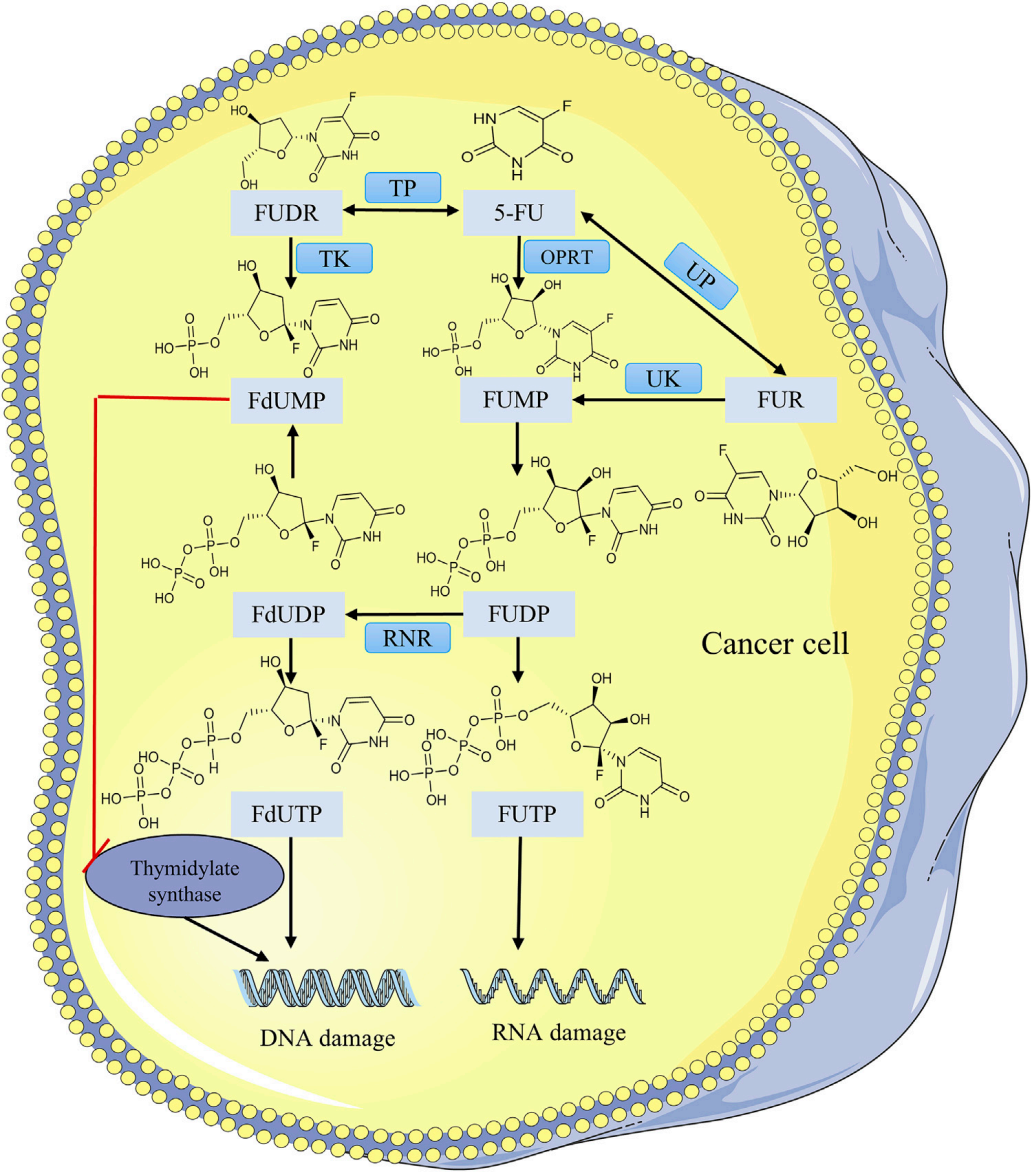
Metabolic action of 5-FU and its active metabolite in the cancer cell.

FOLFIRI is one of the most common standard chemotherapy regimens for the therapy of CRC and consists of three drugs: irinotecan, 5-FU, and leucovorin. 5-FU is a pyrimidine antagonist. The mechanism of 5-FU activation begins with the conversion of 5-FU by the orotate phosphoribosyltransferase (OPRT) and uridine phosphorylase (UP) to fluorouridine monophosphate (FUMP) and fluorouridine (FUR), where FUR is converted indirectly via uridine kinase (UK) to FUMP, which is phosphorylated to fluorouridine diphosphate (FUDP), and then undergoes another phosphorylation process to the active metabolite fluorouridine triphosphate (FUTP) or to fluorodeoxyuridine diphosphate (FdUDP) via ribonucleotide reductase (RNR) (Sethy and Kundu, 2021). FUTP is a fluorinated analog of RNA nucleotides that can be incorrectly doped into the RNA of tumor cells resulting in RNA damage. FdUDP can be phosphorylated or dephosphorylated to generate the active metabolites fluorodeoxyuridine triphosphate (FdUTP) and fluorodeoxyuridine monophosphate (FdUMP). FdUTP is incorrectly doped into the DNA of tumor cells leading to DNA damage (Azwar et al., 2021). Another mechanism of activation is the conversion of 5-FU to fluorodeoxyuridine (FUDR) by thymidine phosphorylase (TP) and then phosphorylation to FdUMP by thymidine kinase (TK) (Figure 4). In conclusion the antitumor activity of 5-FU is the incorporation of its active metabolites into RNA and DNA to interfere with nucleoside metabolism (Vodenkova et al., 2020), and the active metabolite FdUMP irreversibly inhibits thymidylate synthase (TS), leading to DNA damage and tumor cell death. It is often used extensively in the therapy of CRC (Saltz et al., 1996).

Typically, the FOLFIRI regimen is usually administered every 2 weeks for multiple consecutive cycles. In clinical practice, it has been found that patients are prone to congenital or acquired resistance to 5-FU, which means that the efficacy of 5-FU monotherapy is limited, so the effectiveness of clinical treatment is hindered (Longley et al., 2003). 5-FU is typically administered with leucovorin, which increases the affinity of 5-FU for TS and further enhancing the efficacy of 5-FU (Benson and Goldberg, 2003). A meta-analysis also revealed the same results, with the administration of 5-FU and leucovorin increasing response rate (RR) and overall survival (OS) in comparison with 5-FU alone (Thirion et al., 2004). When irinotecan was added to the regimen, it was preferred over 5-FU and leucovorin alone in respect of progression-free survival (PFS), OS and RR, and effectively delayed the progression of cancer (Saltz et al., 2000). Irinotecan has no cross-resistance to 5-FU/leucovorin therapy, which is an essential for its use in combination therapy for CRC.

Douillard et al. (2000) included 387 patients in the study of advanced CRC and randomly divided them into two groups, one of which was applied irinotecan in combination with 5-FU and leucovorin, and the other group applied only 5-FU and leucovorin. According to the findings, the irinotecan had a higher RR (49% vs. 31%, *p* < 0.001), a longer PFS (6.7 vs. 4.4 months, *p* < 0.001), and a longer OS (17.4 vs. 14.1 months, *p* = 0.031) than the non-irinotecan group. While diarrhea and neutropenia in the irinotecan group were more common and severe, several toxic events in grade 3 and 4 were also noticeably more prevalent. Comparison with irinotecan alone, FOLFIRI can reduce the rate of adverse events such as alopecia and diarrhea, and did not affect the effect of clinical treatment. There were no significant differences in terms of change in overall QoL, RR, PFS, or OS between the two groups (Clarke et al., 2011). The key clinical study V308 proved that irinotecan was the first option for first-line treatment of advanced metastatic colorectal cancer (mCRC), which compared the effectiveness of sequential usage of FOLFIRI and FOLFOX6 (oxaliplatin, 5-FU/leucovorin) for the treatment of advanced mCRC. The results showed that FOLFIRI regimen as the first-line treatment option for advanced mCRC had lower overall adverse reactions and was more tolerable (Tournigand et al., 2004).

Subgroup analysis indicated that elderly patients treated with FOLFOXIRI (irinotecan, oxaliplatin, 5-FU/leucovorin) had a higher incidence of severe diarrhea, which did not appear to have a significant benefit in elderly mCRC patients compared with FOLFIRI (Vamvakas et al., 2010). Therefore, FOLFIRI two-drug regimen for first-line chemotherapy is a rational choice in elderly mCRC patients. It is obvious that irinotecan in combination with 5-FU/leucovorin is an advantageous treatment, especially in elderly patients.

In Japan, the cost of chemotherapy with FOLFIRI was lower than that of oxaliplatin-based chemotherapy regimens (FOLFOX, oxaliplatin plus 5-FU/leucovorin), and the use of lower-cost chemotherapy regimens as first-line chemotherapy could reduce the overall cost of the entire chemotherapy course (Yajima et al., 2015). Due to the differences in toxicity between the two regimens, the FOLFIRI regimen is superior to the FOLFOX regimen from the standpoint of long-term health outcomes (Qingwei et al., 2020).

### 2.2 FOLFOXIRI

**FIGURE 5:**
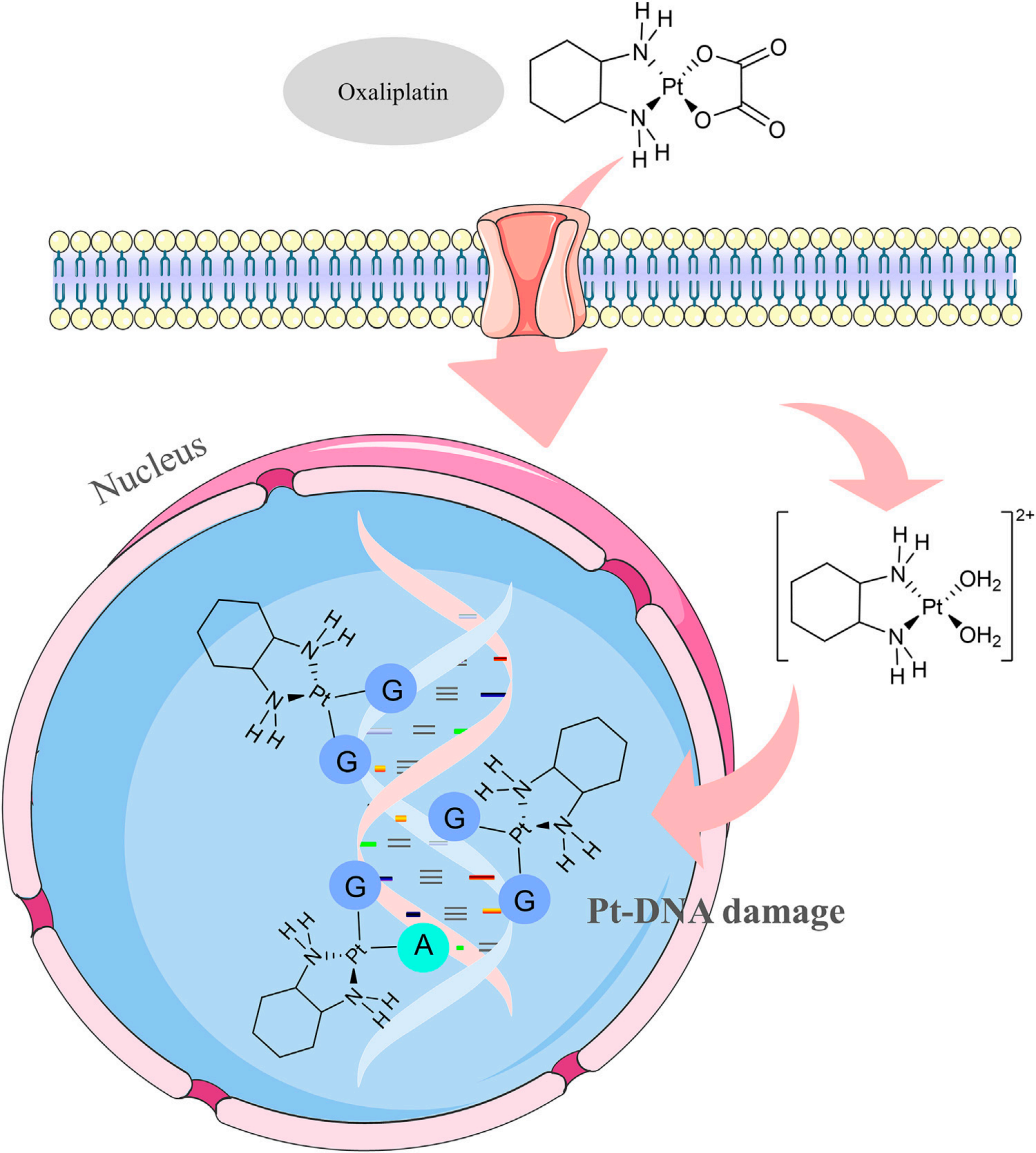
Mechanism of apoptosis in oxaliplatin adduct formation.

The FOLFOXIRI regimen, which combines irinotecan, oxaliplatin, and 5-FU/leucovorin, is a high-intensity chemotherapy regimen. The mechanism of action of oxaliplatin, a cytotoxic drug in this regimen, is not yet fully understood, but according to existing studies, it exerts its cytotoxic effects mainly through DNA damage (O’Dowd et al., 2023). The main target of oxaliplatin is DNA. When oxaliplatin enters the cell, the platinum atoms can combine with the DNA of the tumor cell to form a Pt-DNA adduct, which affects the transcription and replication functions of DNA and ultimately leads to the death of the tumor cell (Szefler and Czeleń, 2023) (Figure 5). The oxaliplatin antitumor process can be divided into four phases, including cellular uptake of the drug, hydration or activation of the drug, DNA platinization, and intracellular processing. The FOLFOXIRI regimen is mainly characterized by good efficacy in terms of objective response rate, PFS, and OS, with manageable and well-tolerated side effects.

At present, FOLFOXIRI regimen has emerged as one of the chemotherapeutic regimens recommended by the guidelines of the Chinese Society of Clinical Oncology (CSCO), European Society for Medical Oncology (ESMO) and National Comprehensive Cancer Network (NCCN) for the treatment of advanced CRC. Initially, there was clinical evidence that concomitant therapy with irinotecan and oxaliplatin for CRC was feasible and effective (Scheithauer et al., 1999). The safety and efficacy of FOLFOXIRI as a first-line therapeutic treatment for mCRC was initially reported in 2002 (Falcone et al., 2002). In an attempt to compare the advantages of the three-agent chemotherapeutic regimen FOLFOXIRI versus the two-agent chemotherapeutic regimen FOLFIRI in the first-line treatment of mCRC, the Gruppo Oncologico Nord Ovest (GONO) carried out a phase III study. The FOLFOXIRI group had a significantly beneficial effect in RR (66% vs. 41%, *p* = 0.0002), OS (22.6 vs. 16.7 months, *p* = 0.032) and median PFS (9.8 vs. 6.9 months, *p* = 0.0006), and adverse events like diarrhea, peripheral nerve reactions and neutropenia were also increased significantly in the FOLFOXIRI group. The occurrence of diarrhea was also significantly increased, and the risk of toxicity was increased with the use of FOLFOXIRI in elderly patients, so the FOLFOXIRI regimen should be used with caution in elderly patients with poor physical condition (Falcone et al., 2007). The HORG study had a total of 283 patients were enrolled, which indicated a significantly more frequent incidence of alopecia, diarrhea, and neurotoxicity in the FOLFOXIRI group in comparison with the FOLFIRI group. The treatment endpoint OS (21.5 vs. 19.5 months, *p* = 0.337) improved in the FOLFOXIRI group as compared with the FOLFIRI group, but the difference was not statistically significant (Souglakos et al., 2006).

Due to poorer tolerability in Asian individuals compared with European populations, the clinical application of the conventional FOLFOIRI regimen in CRC is greatly restricted in China and even throughout the entire Asian region. In China, the dosage of irinotecan in the FOLFOXIRI regimen was adjusted downward from 180 mg/m^2^ to 150–165 mg/m^2^, which is more suitable for the dosage intensity of the Chinese population (Cai et al., 2018). However, the assessment of the efficacy and adverse effects of the modified regimen needs to be confirmed by further clinical studies, due to the limited number of reported cases in this study.

The FOLFOXIRI regimen showed promising outcomes with a PFS of 13.37 ± 9 months, an overall response rate of 79.4%, a significant decrease in the risk of early disease progression, and side effects within the acceptable range for mCRC first-line therapy (Huy et al., 2019). More importantly, the FOLFOXIRI regimen increased the rate of radical surgery for initially unresectable mCRC, and the long-term survival of radically resected patients was particularly notable, with a benefit of 42% and 33% in 5- and 8-year survival rates, respectively (Masi et al., 2009). Compared to the FOLFIRI regimen, FOLFOXIRI provided clinically significant improvements in long-term outcomes, with an absolute benefit in 5-year survival of 7% and improvements in both PFS and OS at long-term follow-up (Masi et al., 2011). Compared to FOLFIRI, the FOLFOXIRI regimen increased drug costs due to the addition of oxaliplatin.

### 2.3 XELIRI

**FIGURE 6:**
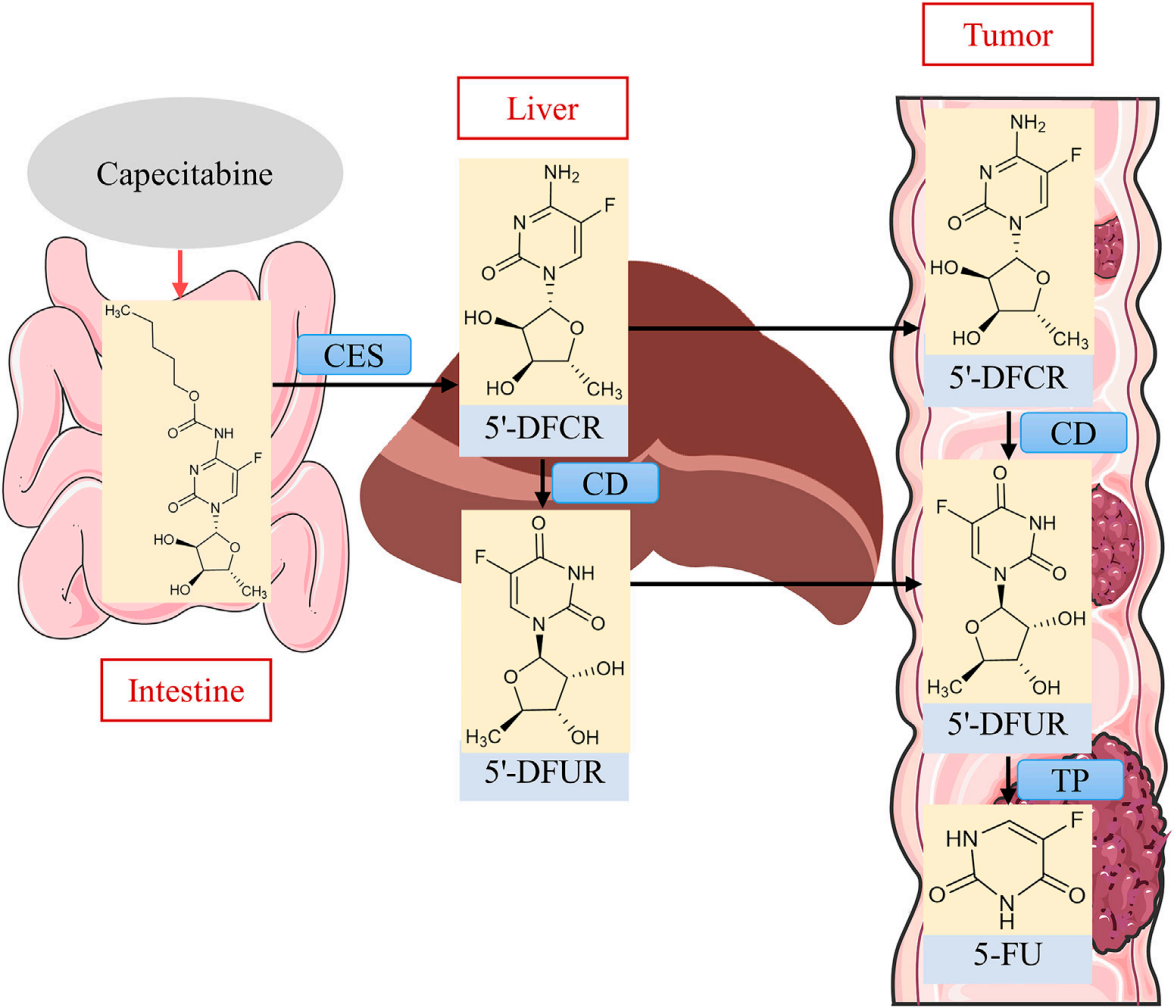
Metabolic activation process of capecitabine. Source of liver illustration: https://scidraw.io/.

The regimen of irinotecan in combination with capecitabine (XELIRI) requires only 2–3 h of infusion every 3 weeks. A phase II single-arm study with mCRC patients showed favorable efficacy and safety (Garcia-Alfonso et al., 2009). Capecitabine is an orally available fluorouracil prodrug with an oral bioavailability of nearly 100%. It has superior safety and convenience and has significant anti-tumor activity (García-Alfonso et al., 2021). Capecitabine is relatively non-cytotoxic *in vitro* and, after oral administration, is readily absorbed through the intestinal mucosa, where it is first catalytically metabolized to 5′-deoxy-5-fluorocytidine (5′-DFCR) in the liver by CES (Walko and Lindley, 2005). Cytidine deaminase (CD) is an enzyme that is found widely and in high concentrations in most tissues, including the liver and tumors (Alzahrani et al., 2023). 5′-DFCR is catalytically converted to 5′-deoxy-fluorouracil (5′-DFUR) by CD in the liver and tumor cells, and finally converted to 5-FU by TP to exert antitumor effects (de With et al., 2023) (Figure 6). The last metabolic step is thought to occur preferentially in tumor tissues because TP activity is very low in normal tissues, reducing the exposure of 5-FU in normal tissues, whereas the concentration of this enzyme is markedly elevated in tumor tissues, and thus capecitabine is effective in enhancing antitumor effects and reducing systemic toxicity (Pentheroudakis and Twelves, 2002).

When neoadjuvant preoperative radiotherapy of irinotecan in combination with capecitabine was guided by the UGT1A1 gene, increasing the dose of irinotecan can enhance the pathologic complete response rate from 15% to 30%, which may become an improved strategy for locally advanced CRC patients to achieve better tumor regression, significantly improve the clinical effective rate, and the toxicity and side effects are within the acceptable range (Zhu et al., 2020). A meta-analysis that compared the efficacy of the XELIRI and FOLFIRI regimens for the first-line therapy of mCRC indicated no apparent distinction in terms of OS, overall response rate or PFS, and the safety profiles of the two regimens were comparable (Guo et al., 2014). The combined treatment of irinotecan and capecitabine is logical, which can not only maintain the efficacy of the combination regimen, but also take advantage of the convenience of capecitabine in the treatment process. From a long-term safety perspective, the XELIRI regimen increased gastrointestinal toxicity compared to FOLFIRI (Montagnani et al., 2010).

FOLFIRI regimen requires 46 h of infusion every 2 weeks, and patients need to undergo central venous intubation before treatment, which is inconvenient to use. Based on the limitations of the FOLFIRI regimen, the toxicity caused by the standard dose of XELIRI and the difference in tolerance among different populations (Haller et al., 2006; Fuchs et al., 2007). The AXEPT study, the first large multicenter randomized controlled phase III study of the modified XELIRI (mXELIRI, capecitabine plus irinotecan) regimen compared to the FOLFIRI regimen, supported the use of the mXELIRI regimen as an alternative second-line treatment option for patients with mCRC (Xu et al., 2018). A cost-benefit analysis showed that compared with FOLFIRI, mXELIRI regimen is a cost-effective second-line treatment for mCRC in China (Wu et al., 2020). According to the AXEPT study, OS with mXELIRI in combination with or without bevacizumab (16.8 vs. 15.4 months, *p* < 0.0001) was no less than with the standard FOLFIRI regimen. Regarding safety, the incidence of neutropenia, the most prevalent grade 3–4 adverse event, was considerably lower in the mXELIRI group in 17% (52 of 310 patients) than in the FOLFIRI group in 43% (133 of 310 patients). Grade 3–4 diarrhea in the mXELIRI arm than the incidence in most previous full-dose XELIRI trials (Koopman et al., 2007; Köhne et al., 2008; Souglakos et al., 2012). The incidence of serious adverse incidents was 15% in the mXELIRI group, whereas it was 20% in the FOLFIRI group. There was no discernible difference in PFS between the two groups. According to the overall findings, modified XELIRI may be an efficacious, tolerable, and more convenient therapeutic option replace FOLFIRI as a standard second-line backbone regimen for Asian mCRC patients. Considering the results of this study, the modified XELIRI regimen will be expected to replace the FOLFIRI regimen as the new standard chemotherapy regimen for advanced CRC patients worldwide, especially in Asia, changing the current clinical practice.

### 2.4 Bevacizumab

**FIGURE 7:**
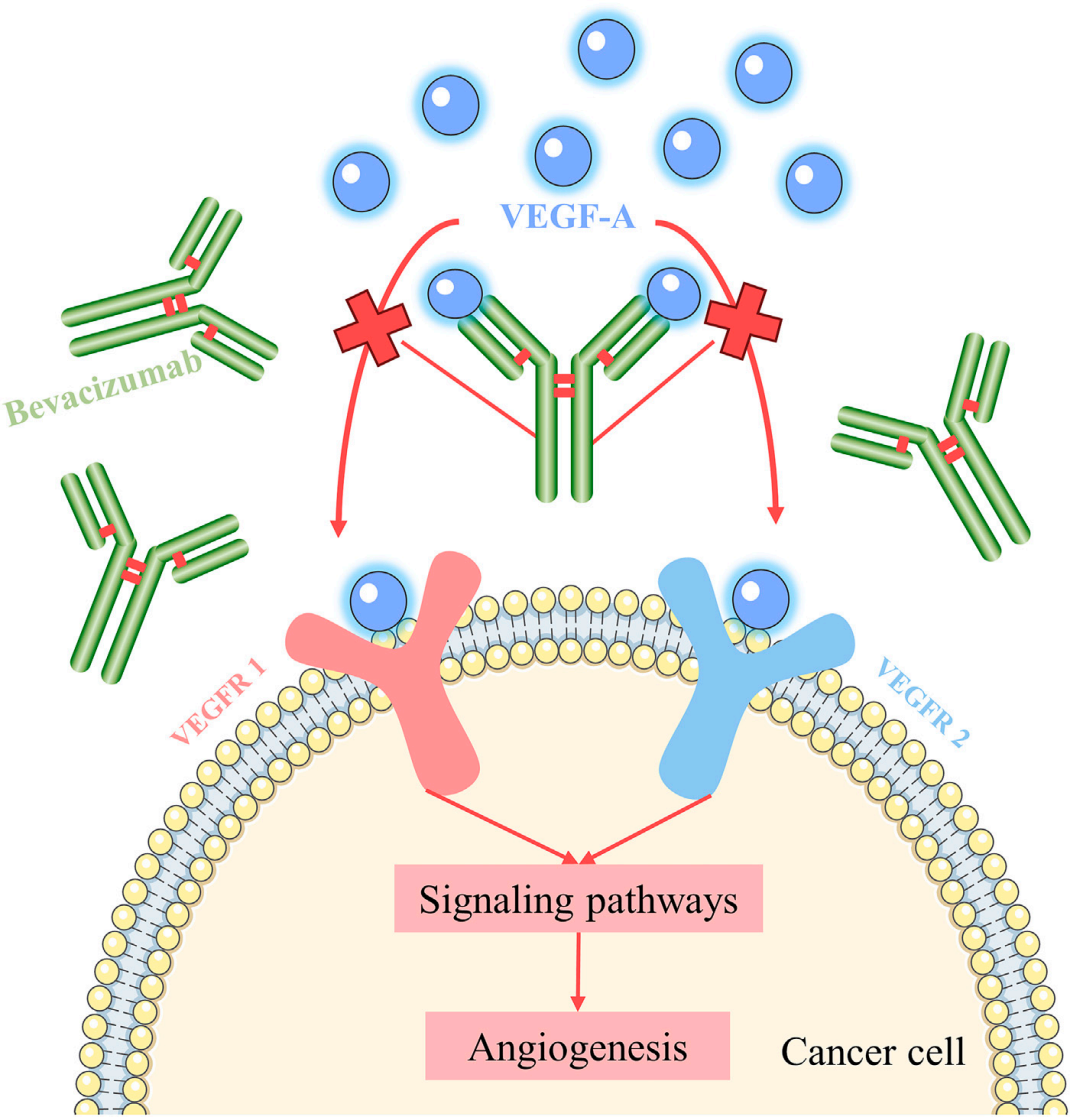
Mechanism of action of bevacizumab.

The US Food and Drug (FDA) authorized bevacizumab as the first targeted drug in 2004 for CRC treatment, signaling the start of a new series of anti-cancer therapies (Heinemann and Hoff, 2010). Bevacizumab is still the most widely used and characterized anti-vascular endothelial growth factor monoclonal antibody. VEGF is the most important angiogenic player, and VEGF stimulates endothelial cell proliferation and survival and increases vascular permeability, thereby supporting the metabolic demands of tumor growth (Apte et al., 2019). Among them, Vascular endothelial growth factor A (VEGF-A) is a major mediator of tumor angiogenesis and induces angiogenesis through direct action on endothelial cells. VEGF-A activates VEGF signaling in endothelial cells by binding to VEGF rceptor-1 (VEGFR-1) and VEGF receptor-2 (VEGFR-2) (Méndez-Valdés et al., 2023). VEGFR-2 is mainly involved in tumor pathological processes such as tumor angiogenesis and is the most important inducer, and VEGFR-1 plays an important role mainly in tumor growth and progressive inflammatory processes (Melincovici et al., 2018). Bevacizumab exerts anti-tumor effects by binding to VEGF-A, preventing VEGF-A from interacting with VEGFR-1 and VEGFR-2, blocking the signaling pathway of angiogenesis, and inhibiting the formation of tumor neovasculature, thus inhibiting the growth of tumor cells (Gerber and Ferrara, 2005) (Figure 7).

Bevacizumab is commonly administered as an addition to standard chemotherapy regimens to provide an effective therapeutic option for a spectrum of CRC patients with a poor prognosis. For induction and maintenance therapy, the combination of bevacizumab with chemotherapeutic drugs is advised. The inclusion of bevacizumab to irinotecan-based backbone chemotherapy regimens is a standard choice for first-line treatment for mCRC patients (Garcia et al., 2020). However, adverse events of proteinuria and thromboembolism occurred in mCRC patients chronically treated with bevacizumab when the dose of the drug exceeded the threshold dose (Fukuda et al., 2023). Adding bevacizumab to first- and second-line therapy increased costs by $60,000 and $40,000, respectively, and prolonged median OS by 6 weeks for both, but bevacizumab was more cost-effective in second-line therapy due to the shorter duration of therapy (Goldstein et al., 2015, 2017).

With the addition of bevacizumab to FOLFIRI significantly improved RR (44.8% vs. 34.8%, *p* = 0.004), as well as prolonged median PFS (10.6 vs. 6.2 months, *p* < 0.001) and OS (20.3 vs. 15.6 months, *p* = 0.00003) in AVF2107g, the first phase 3 study to evaluate the effect of bevacizumab in the treatment of first-line mCRC patients (Hurwitz et al., 2004). Bevacizumab and two irinotecan-based backbone chemotherapy regimens FOLIRI and XELIRI were useful first-line therapies for the treatment of mCRC patients, with similar safety profiles and expected endpoints, according to a randomized, non-controlled study (Ducreux et al., 2013). A meta-analysis published in 2020 reviewed 11 studies including 5632 patients to compare the effectiveness and safety of bevacizumab with irinotecan-based or oxaliplatin-based dual-backbone chemotherapy as first-line treatment options for mCRC. The findings showed that bevacizumab and irinotecan-based chemotherapy had more advantages in improving PFS. The study recommended that bevacizumab plus irinotecan-based backbone chemotherapy is the first-line therapeutic choice for prolonging PFS (Ren et al., 2021).

The TRIBE-2 study demonstrated that FOLFOXIRI plus bevacizumab improved OS (29.8 vs. 25.8 months, *p* = 0.03) in patients with mCRC in comparison with FOLFIRI plus bevacizumab (Cremolini et al., 2015). A randomized controlled trials found that FOLFOXIRI combined with bevacizumab led to an improved prognosis in mCRC patients, but the frequency of adverse events was also relatively increased (Loupakis et al., 2014). These provide compelling support for the first-line treatment of irinotecan-based three-drug regimen FOLFOXIRI combined with bevacizumab. For patients with mCRC who are generally in good condition, the strategy of FOLFOXIRI palliative care, sequential maintenance therapy, and treatment progression followed by FOLFOXIRI plus bevacizumab can be used (Cremolini et al., 2020). At present, the TRIBE-C (NCT04230187) study is in progress to assess whether the Chinese modified version of FOLFOXIRI combined with bevacizumab for advanced CRC can further improve the efficacy, safety and feasibility compared with the traditional regimen combined with bevacizumab.

### 2.5 Panitumumab

**FIGURE 8:**
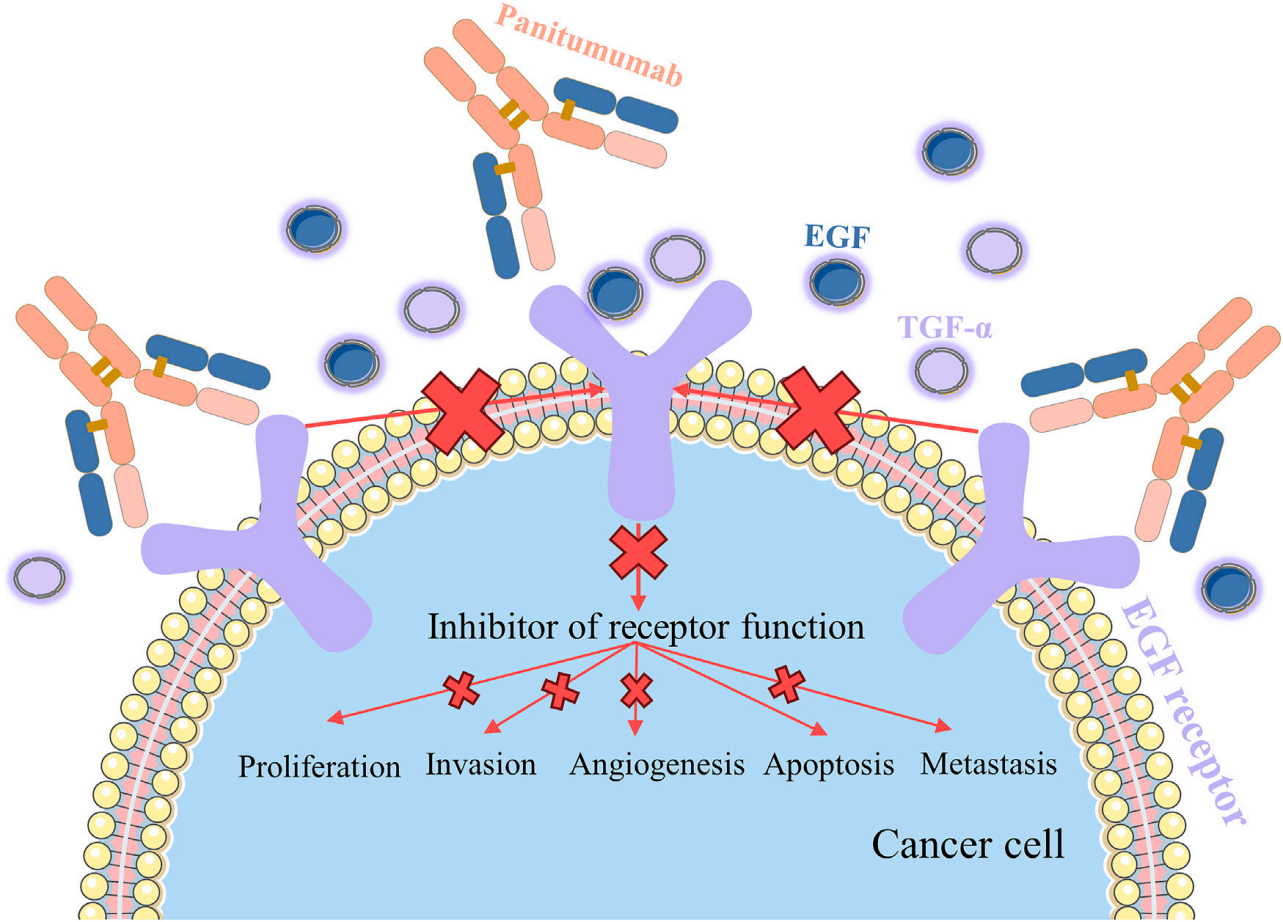
Mechanism of action of panitumumab.

Panitumumab, an epidermal growth factor receptor (EGFR) antagonist, is indicated for the treatment of EGFR-expressing mCRC (Hoy and Wagstaff, 2006). EGFR is highly or aberrantly expressed in a variety of cancers, stimulates proliferation, angiogenesis, and metastasis, and protects tumor cells from apoptosis (Uribe et al., 2021). Panitumumab is the first fully human monoclonal antibody approved for the treatment of CRC, so it is less likely to induce an immunogenic response (Ohishi et al., 2023). Panitumumab is an IgG2 monoclonal antibody that has a stronger affinity for EGFR than cetuximab, binds more readily to EGFR, and effectively blocks the binding of epidermal growth factor (EGF) or transforming growth factor-alpha (TGF-α) ligands to EGFR (Rastin et al., 2024) (Figure 8). Panitumumab acts as a functional antagonist of EGF and TGF-α ligands, leading to the internalization and degradation of antibody-receptor complexes, thereby inhibiting the EGFR-mediated signaling pathway, and the signaling blockage leads to the inhibition of tumor cell division, which inhibits tumor growth, metastasis, and angiogenesis and promotes apoptosis of tumor cells (Adebayo et al., 2023).

Panitumumab may also play a direct or indirect antitumor role by enhancing the cytotoxic effects of other drugs and may be used as monotherapy for irinotecan-resistant tumors or in combination with irinotecan for the treatment of mCRC. Panitumumab plus irinotecan appeared to be no less effective than cetuximab combined with irinotecan in the therapy of *KRAS* wild-type exon 2 mCRC patients (Daisuke et al., 2020). Clinical studies have indicated that cetuximab decreased efficacy in patients previously treated with bevacizumab (Sato et al., 2015). In contrast, panitumumab was more effective than cetuximab individuals previously treated with bevacizumab (Price et al., 2016). Panitumumab is used in combination regimens and has been studied for potential interactions with chemotherapeutic agents. The pharmacokinetic profile of irinotecan co-administered with or without panitumumab is almost identical. Panitumumab does not effect on the pharmacokinetics of irinotecan or there are synergistic effects (Yang et al., 2013).

The PICCOLO trial included 460 advanced *KRAS* wild-type CRC patients that had not been treated previously with EGFR-targeted agents; the PFS in the panitumumab plus irinotecan group was markedly superior to that of the irinotecan group alone, and there was no differentiation in the OS between the two groups, with the combination of irinotecan plus panitumumab not improving the OS in patients with *KRAS* wild-type tumors (Seymour et al., 2013). In treatment-refractory mCRC, treatment is palliative rather than curative, and the main goal is to maximize patient survival and maintain QoL. In *KRAS* wild-type patients, the irinotecan-based chemotherapy regimen FOLFIRI combined with panitumumab achieved a median PFS of 5.9 months, which was a significant increase versus 3.9 months in the FOLFIRI group (*p* = 0.004). With the administration of panitumumab, the RR increased from 10% to 35% although the OS did not dramatically increase (Peeters et al., 2010). The combination of FOLFIR with panitumumab was seen to be effective. In the presence of irinotecan dose reduction, panitumumab in combination with FOLFOXIRI reduced irinotecan-induced diarrhea (Fornaro et al., 2013). In addition, the JACCRO CC-14 study supported that the FOLFOXIRI plus panitumumab regimen was well tolerated in *RAS* wild-type mCRC patients with irinotecan dose reduction (Satake et al., 2018). With an objective response rate of 87.3% (87.3% vs. 60.06%, *p* = 0.004) and a secondary resection rate of metastases with panitumumab of33.3% (33.3% vs. 12.1%, *p* = 0.02), the combination of panitumumab with the modified FOLFOXIRI regimen improved the objective response rate and secondary resection rate of metastases in *RAS* wild-type mCRC patients (Modest et al., 2019). It is clear that the efficacy of panitumumab in a highly active chemotherapy backbone is not reduced.

A case of a patient with metastatic chemotherapy-refractory CRC who was treated with panitumumab monotherapy for more than 65 months was reported in 2010, with sustained efficacy over a prolonged period of time, significant prolongation of PFS, and persistent and generally stable skin toxicity (Seront et al., 2010). In the *RAS* wild-type subgroup of mCRC, the use of anti-EGFR (panitumumab or cetuximab) in first-line therapy was more favorable cost-effective compared to anti-VEGF (bevacizumab) (Koilakou and Petrou, 2021).

The chemotherapeutic regimen of panitumumab combined with the cytotoxic drug irinotecan prolonged the PFS or the toxicity of the drug was tolerable in *RAS* wild-type mCRC patients, but there are no remarkable results in OS. Overall, irinotecan combined with panitumumab is positive, safe and feasible as a salvage treatment.

### 2.6 Cetuximab

**FIGURE 9:**
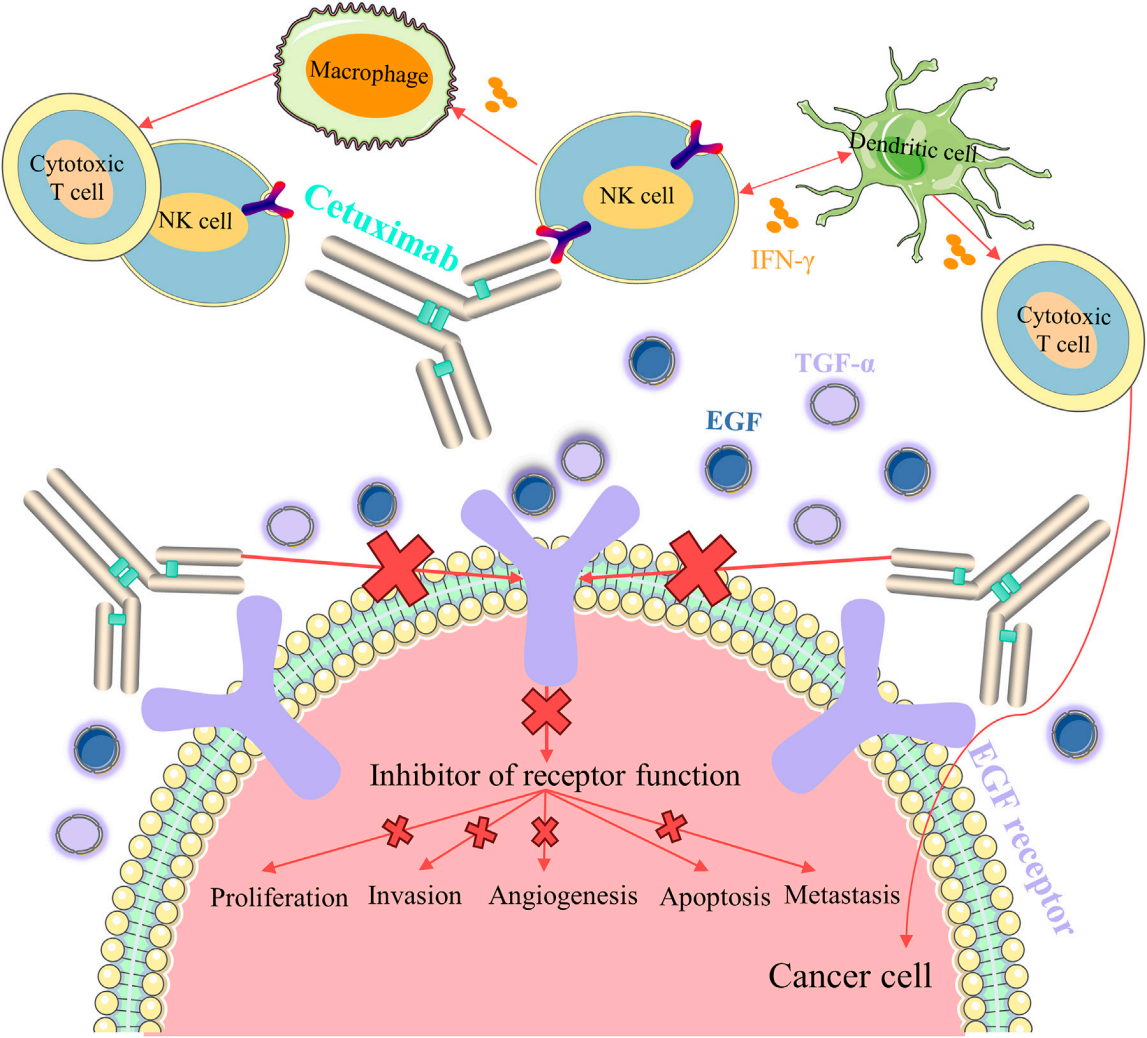
Mechanism of cetuximab action and mediated immune activity.

With solid preclinical evidence, cetuximab was considered as the first mouse-human monoclonal antibody targeting EGFR in 1995, and the FDA authorized cetuximab for the therapeutic use of mCRC in 2004 (Mendelsohn et al., 2015). Cetuximab, similar to panitumumab, is an EGFR inhibitor that suppresses tumor cell growth and metastasis by inhibiting the EGFR signaling pathway. However, unlike panitumumab, cetuximab has antibody-dependent cell mediated cytotoxicity (ADCC) effect that triggers immune anti-tumor effects (García-Foncillas et al., 2019). Natural killer (NK) cells are activated by binding to cetuximab uploaded onto the EGFR, and released interferon-gamma (IFN-γ) activates dendritic cells, which further activates NK cells (Xiong et al., 2023) (Figure 9). Cetuximab-induced ADCC releases antigens, which are captured by activated dendritic cells and presented to T cells, and in turn, mature dendritic cells are able to activate a variety of additional immunogenic processes, including antigen presentation to cytotoxic T cells and further activation of NK cells (Kasi et al., 2023). IFN-γ mediated crosstalk between macrophages and other immune cells is critical for bringing additional active cytotoxic T cells into the intra-tumor space, and these neurotoxic T cells can subsequently undergo lysogenic activity against tumor cells, resulting in the production of additional tumor antigens and further stimulation of long-term immune responses (Ferris et al., 2018).

To research the therapeutic effectiveness of cetuximab plus irinotecan and cetuximab monotherapy in the treatment of patients with refractory CRC, the researchers recruited 329 patients. The results indicated that the combined treatment group had a significantly greater RR (22.9% vs. 10.8%, *p* = 0.007) than the monotherapy group and a significantly prolonged median time to progression (4.1 vs. 1.5 months, *p* < 0.001) (Cunningham et al., 2004). It has been suggested that cetuximab can restore the sensitivity of irinotecan. In the EPIC trial, it was showed that the additional administration of cetuximab to irinotecan cloud improve PFS and RR in mCRC patients, with a QoL superior to that of irinotecan alone and without causing a significant increase in toxicity (Sobrero et al., 2008). The AGITG ICECREAM trial confirmed that irinotecan combined with cetuximab had significant benefits in *RAS* wild-type mCRC patients compared with cetuximab alone. Cetuximab in combination with irinotecan improved the RR and delay the disease progression of *RAS* wild-type CRC resistant to irinotecan (Shapiro et al., 2018). This retrospective analysis showed that irinotecan combined with cetuximab improved objective response rate, PFS, and QoL in *RAS* wild-type mCRC patients (Sobrero et al., 2021). The combination of irinotecan and cetuximab appears to be more advantageous for the treatment of *RAS* wild-type mCRC than irinotecan or cetuximab monotherapy.

The FIRE-3 research found that in *KRAS* exon 2 wild-type mCRC patients, cetuximab in addition to with the standard irinotecan-based chemotherapy regimen FOLFIRI was superior to bevacizumab combined with FOLFIRI, and patients achieved higher objective response rate and prolonged OS (Heinemann et al., 2014). The AIO KRK-0306 trial further supported that FOLFIRI plus cetuximab was more beneficial than FOFLIRI plus bevacizumab in the treating *RAS* wild-type mCRC (Stintzing et al., 2016). The POCHER study indicated that for *RAS/BRAF* wild-type patients, FOLFOXIRI in combination with cetuximab treated patients with initially unresectable CRC liver metastases with a resection rate of up to 60% (Garufi et al., 2010). The MACBETH study demonstrated that overall response rate of 71.6% for FOLFOXIRI in combination with cetuximab in *RAS/BARF* wild-type patients (Cremolini et al., 2018). A phase Ib study in Japan also revealed that combination of cetuximab and FOLFOXIRI had controllable toxicity and good efficacy in *RAS* wild-type mCRC (Kadowaki et al., 2021).

A retrospective subgroup analysis showed that cetuximab in combination with irinotecan-based FOLFIRI is an ideal regimen to promote an increase in objective response rate, with higher objective response rate promoting resectability, which in turn contributes to improved long-term survival (Köhne et al., 2016). For unselected patients with advanced CRC, the incremental cost of cetuximab is high, and when limited to *KRAS* wild-type patients, the incremental cost is low (Mittmann et al., 2009). In *RAS* wild-type mCRC patients, the use of cetuximab added to irinotecan-based backbone chemotherapy regimens is feasible, demonstrating positive antitumor activity. However, further controlled trials are required to ascertain whether it prolongs patient survival.

### 2.7 PHY-906

PHY-906 is derived from a traditional formula used for thousands of years, Huang Qin Tang (HQT), a traditional formula used for thousands of years in Zhang Zhongjing’s The Treatise on Typhoid Fever of the Eastern Han Dynasty, which is primarily used for treating gastrointestinal disorders including diarrhea, abdominal cramps, nausea and vomiting (Qin et al., 2022). It is composed of *Paeonia lactiflora* Pall, *Glycyrrhiza uralensis* Fisch, *Scutellaria baicalensis* Georgi and *Ziziphus jujuba* Mill (Tilton et al., 2010). PHY-906, a natural mixture extracted from these four herbs, is a unique novel anti-tumor drug candidate developed based on an integrated systems biology approach. However, it differs from HQT in terms of drug source, biological activity, and pharmacodynamic composition (Liu and Cheng, 2012). PHY-906 is a kind of strictly in accordance with the Current Good Manufacture Practices (cGMP) specifications, each production step has strict standard operating procedures, which fundamentally ensures the consistency of drug quality (Lam et al., 2018).

**FIGURE 10:**
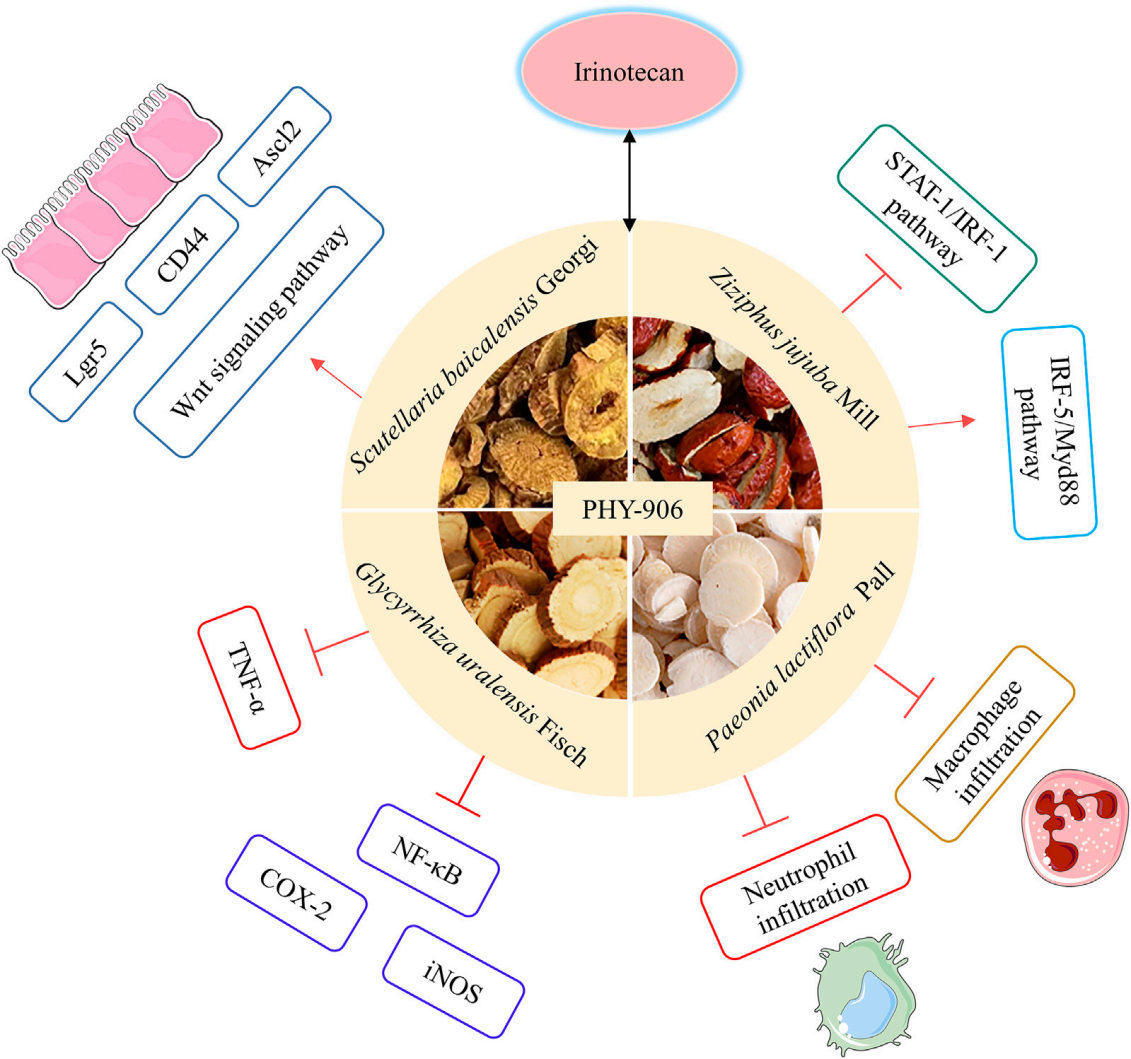
Possible mechanisms by which PHY906 counteracts irinotecan toxicity.

Diarrhea is one of the most important dose-limiting toxic reactions of irinotecan in the treatment of CRC, and the opioid receptor agonist loperamide is commonly used clinically to alleviate the diarrhea induced by irinotecan without reaping satisfactory results (Abigerges et al., 1994). According to preclinical studies, PHY-906 enhances the anticancer activity of irinotecan while decreasing irinotecan-induced weight loss and mortality. PHY-906 reduces irinotecan-induced inflammation by decreasing neutrophil or macrophage infiltration, decreasing the expression of tumor necrosis factor-alpha (TNF-α) in the intestines, and decreasing plasma concentrations of proinflammatory cytokines. A variety of PHY-906's chemical constituents or metabolites can effectively inhibit the NF-κB pathway and directly inhibit cyclooxygenase-2 (COX-2) and inducible nitric oxide synthase (iNOS) activity to mediate this mechanism. PHY-906 also promotes the growth of intestinal progenitor and stem cells through activation of the Wnt signaling, thereby accelerating the regeneration and recovery of damaged gastrointestinal tissues (Lam et al., 2010). When PHY-906 is administered with irinotecan, PHY-906 causes activation of the IRF-5/Myd88 pathway and reverses the inhibition of the STAT-1/IRF-1 pathway, with significant immune effects (Wang et al., 2011) (Figure 10). Researchers screened baicalin, baicalein, glycyrrhizic acid and wogonin in PHY-906 through *in vivo* and *in vitro* assays and identified them as key bioactive constituents with the ability to enhance the anticancer effects of irinotecan while reducing the intestinal toxicity induced by irinotecan (Dou-Dou et al., 2021).

The combination of PHY-906 with irinotecan does not change the pharmacokinetic parameters of irinotecan or influence the transformation of irinotecan to its active metabolite SN-38. A double-blind, randomized, dose-escalation, placebo-controlled phase I trial included 17 advanced CRC patients (Kummar et al., 2011). Investigators found that during irinotecan in combination with PHY-906 chemotherapy, patients had no treatment-related, life-threatening grade IV adverse incidents, and the general incidence of grade 3/4 diarrhea, as well as the frequency and severity of patient vomiting, was low. In contrast, there were two life-threatening grade IV adverse cases in the placebo group, including neutropenia and gastrointestinal bleeding. The Phase I/IIA clinical study used a multicenter, double-blind, randomized, placebo-controlled, dose-escalation cross-over clinical trial methodology (Farrell and Kummar, 2003), in which patients with advanced CRC were randomized into 2 groups, both of which were treated with FOLFIRI or irinotecan, with group 1 receiving PHY-906 in the first cycle and placebo in the second cycle, and group 2 receiving the opposite. The results of the study showed that PHY-906 could improve the anti-tumor efficacy of chemotherapeutic drugs, significantly reduce the intestinal adverse effects caused by irinotecan, especially diarrhea, and PHY-906 did not adversely impact the anti-tumor activity of irinotecan. It is suggested that PHY-906 can reduce the adverse reactions caused by irinotecan without affecting the anti-tumor activity of irinotecan. Based on the interaction of irinotecan combined with PHY-906 in the inflammatory process of the tumor microenvironment, it was found that PHY-906 could enhance the anti-tumor activity of irinotecan by promoting apoptosis of tumor cells and polarization of macrophages into M1-type macrophages. Most of the herbal remedies used in traditional Chinese medicine have been developed for long-term use, and they have certain long-term survival benefits (Rieder, 2015). Although clinical studies have shown promising results with PHY-906, there is insufficient evidence to conclude that PHY-906 is cost-effective in providing symptomatic relief for CRC survivors.

### 2.8 Silymarin

Silymarin is a natural flavonoid found in the perennial herb artichoke, and the main active ingredient of silymarin is silybin (Koushki et al., 2023). Silymarin is non-toxic even at very high doses and can disrupt with cycle regulation, apoptosis, angiogenesis, and expression of proteins associated with multidrug resistance (Koltai and Fliegel, 2022). On the contrary, the good antioxidant activity of silymarin makes it a useful agent for the prevention of cancer and has a certain place in cancer therapy (Wang et al., 2023).

**FIGURE 11:**
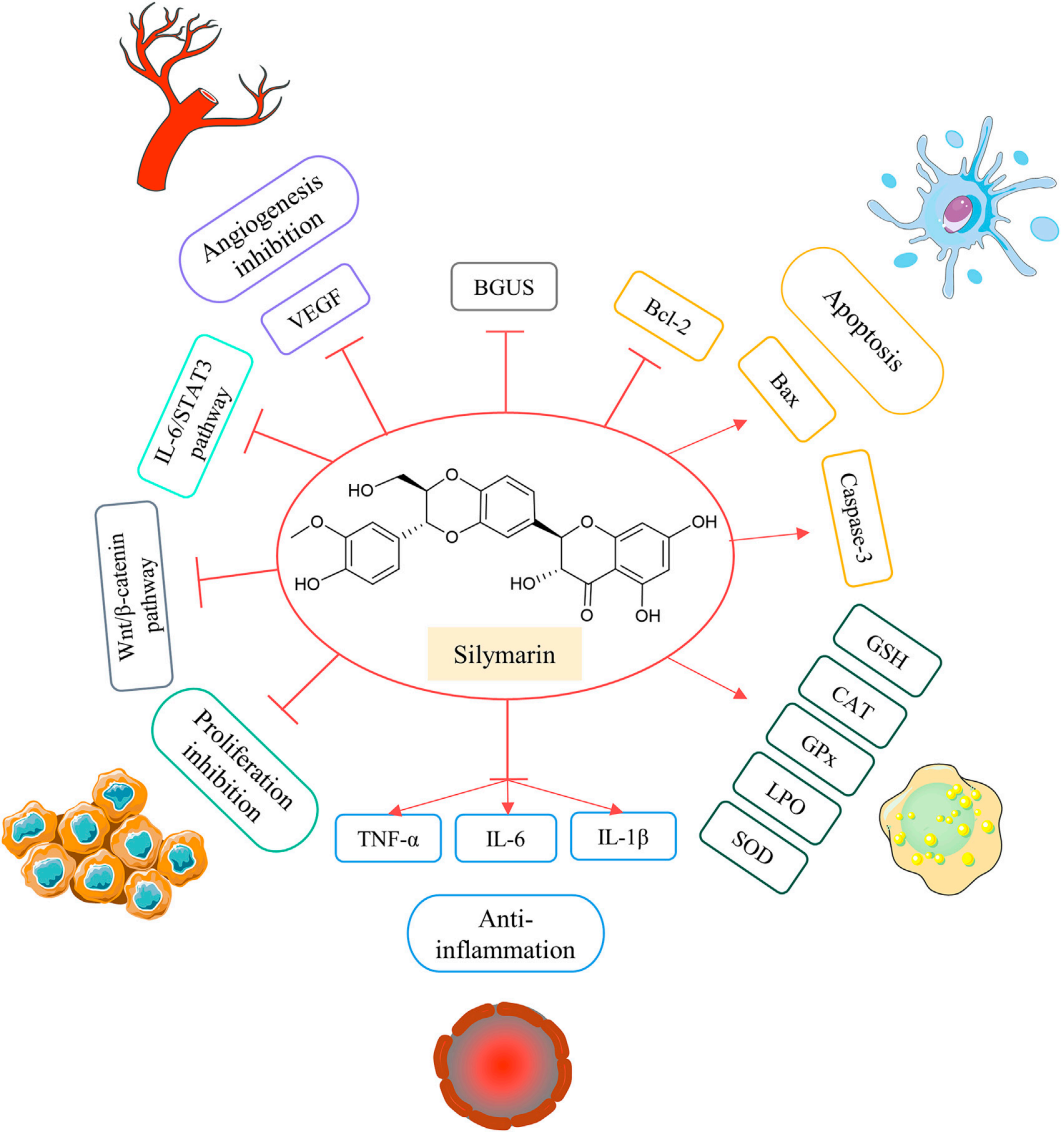
Possible anti-colorectal cancer effects of silymarin.

According to preclinical studies, silymarin leads to inhibition of Wnt signaling in human CRC cells through downregulation of β-catenin and TCF4 (Eo et al., 2016). *In vitro* experiments confirmed the strong anti-angiogenic effect and anti-proliferative activity of silymarin on LoVo CRC cell lines (Yang et al., 2003; Colombo et al., 2011). The anti-CRC activity of the silymarin-oxidized azoxymethane (AOM)-induced colon cancer model was also validated in that silymarin reduced the number of aberrant crypt foci (ACF) in the colon, and dietary intake of silymarin reduced colonic BGUS activity (Kohno et al., 2002). Also in the AOM colitis-associated cancer model, the researchers found that silymarin, the active ingredient of silymarin, significantly downregulated interleukin-6 (IL-6), that interleukin-1beta (IL-1β) and TNF-α could act as prophylactic agents for colitis-associated cancers, and that silymarin prevented colitis-associated tumorigenesis in mice by inhibiting IL-6/STAT3 signaling pathway (Zheng et al., 2018). Silymarin also inhibits 1,2-dimethylhydrazine (DMH)-induced colon carcinogenesis by modulating the activity of intestinal microbial enzymes, the level of colonic oxidative stress, and the Wnt/β-catenin signaling to exert its antiproliferative effects (Sangeetha et al., 2009; 2010b; 2010a). Silymarin significantly reduces survival and induces apoptosis and autophagy in mouse CT26 CRC cells by up-regulating Bax and Caspase-3 and down-regulating Bcl-2 expression (Sameri et al., 2021) (Figure 11).

Silymarin does not work as a stand-alone anticancer agent and may be an important factor in multidrug combination anticancer regimens. For patients with mCRC, the irinotecan-based FOLFIRI chemotherapy regimen has demonstrated its benefit in first-line treatment; however, this regimen causes significant intestinal toxicity, which directly affects the clinical treatment effect of patients with CRC. The main reason for the intestinal reaction caused by irinotecan and metabolites excreted into the intestines via the biliary are converted in the action of BGUS to active SN-38, which leads to delayed diarrhea (Cao, 2019; Huang and Zhu, 2021). Animal experiments inhibited chemically evoked colon cancer in rats by dietary intake of silymarin (Kohno et al., 2002). Silymarin and its components may decrease risk factors for colon cancer by blocking hydrolysis of glucuronides in metabolites (Kim et al., 1994). Silymarin may serve as a good adjuvant to irinotecan in combination, which opens up the possibility of irinotecan in combination with silymarin for treating CRC. A prospective open-label pilot clinical trial evaluated silymarin as a supplement in irinotecan-based therapy in mCRC patients (Chang et al., 2021). FOLFIRI plus the targeted agent bevacizumab was administered to 35 patients with mCRC as the group of control, and 35 patients in the study group received oral silymarin capsules on the basis of control group chemotherapy. With the administration of silymarin capsules, the occurrence of toxic incidents was lower in the study group, with lower rates of nausea (27.0% vs. 40.2%, *p* = 0.005) and diarrhea (5.4% vs. 14.6%, *p* = 0.002). The addition of silymarin slowed the incidence of nausea and diarrhea in patients. Neither short-term (4 days) nor longer-term (12 days) ingestion of silymarin had a significant effect on the clearance of irinotecan (van Erp et al., 2005).

Silymarin has shown superiority as an effective and well-tolerated supplement in irinotecan-based chemotherapy regimens, and silymarin may be a safe and effective option as a complementary therapy to irinotecan-containing chemotherapy regimens. Coupled with the superior cost-effectiveness of silymarin as a natural product, the use of silymarin to enhance the effectiveness of existing anticancer drugs is a very promising approach (Dheeraj et al., 2018).

## 3 Conclusion

The chemotherapy strategy for CRC is determined based on the patient’s condition, and survival outcome will be the primary determinant of the choice of any one strategy. Irinotecan has promising antitumor activity in CRC, and irinotecan-based regimens have a survival advantage and can enhance the effect of irinotecan-based chemotherapy. Various combinations of irinotecan-based combinations are recommended by NCCN guidelines and CSCO guidelines for chemotherapy of different types of colorectal cancer. The current irinotecan-based combination chemotherapy regimens, FOLFIRI, FOLFOXIRI and XELIRI, are also the backbone of clinical chemotherapy for CRC. The role that the combinations play in the treatment process is in part dependent on the irinotecan backbone-based chemotherapy regimen used. With the development and marketing of a variety of molecular targeted drugs that include bevacizumab, cetuximab, and panitumumab, targeted therapies in combination with chemotherapy provide a more efficient and targeted treatment option for CRC. Irinotecan in combination with different targeted agents has its own specific adverse events, and the choice of the optimal combination and sequencing depends on the pre-molecular characteristics of CRC.

Herbal medicines such as silymarin capsules and PHY-906 have had a significant impact on CRC as complementary and alternative therapies. Irinotecan-based regimens combining herbs have shown superior performance in improving patients’ QoL. The chemical composition in herbal medicines is diverse and mechanistically complex, and further research should concentrate on characterizing bioactive compounds with therapeutic effects and delving into their mechanisms of action. Many of the clinical trials were poorly designed, included small patient samples, had poor methodological control, short treatment and follow-up periods, lacked a more rigorous and robust clinical rationale, and the results obtained need to be treated with caution. In the future, larger and more methodologically justified randomized controlled trials should be conducted, otherwise it will be difficult for clinical studies to demonstrate the benefits exerted by herbal medicines.

Toxic reactions to irinotecan are reversible, non-cumulative and controllable. Irinotecan-based chemotherapy regimens can be used at various times during CRC treatment, whether during neoadjuvant, transformational or palliative care and play an irreplaceable and important role. We expect that more personalized irinotecan-based therapies will be optimized or developed, enabling patients to achieve longer survival with fewer adverse effects.

## 4 Future perspective

In order to explore the prospects for further use of irinotecan, several studies of irinotecan and its chemotherapy combinations in the treatment of CRC are still underway. The TRIPLETE study was designed to explore whether the first-line treatment of patients with *RAS/BRAF* wild-type mCRC with the three-agent combination of panitumumab in combination with irinotecan, mFOLFOXIRI, would result in increased efficacy compared to the mFOLFOX6 regimen. The primary study endpoint was not met, and the combination of mFOLFOXIRI with panitumumab did not provide a therapeutic benefit and increased the incidence of gastrointestinal toxicity (Cremolini et al., 2022). The triple combination of anti-EGFR monoclonal antibody combined with irinotecan may provide tumor remission and survival benefit for *RAS* wild-type mCRC. In contrast, the TRIPLETE study showed that irinotecan-based triple-agent combination regimens did not show added value versus mFOLFOX6 chemotherapy when compared to mFOLFOX6 chemotherapy in clinically selected molecularly based populations, which is different from previous knowledge. This suggests that the three-agent chemotherapy regimen of irinotecan is not inapplicable to the population, and that further refinement of the population is needed if better efficacy is to be achieved by increasing the intensity of chemotherapy. The three-agent chemotherapy regimen of irinotecan can be tried in young patients with significant symptomatic disease, patients with insensitivity to chemotherapeutic agents, patients with large tumor loads, and patients with advanced colorectal cancer with peritoneal or multiple metastases. In clinical practice, investigators still need to choose the appropriate regimen from patient characteristics, tumor characteristics and other multifactorial considerations to obtain better survival outcomes.

## Figures and Tables

**Table:** 

**ACF**	Aberrant crypt foci
**ADCC**	Antibody-dependent cellular cytotoxicity
**AOM**	Azoxymethane
**APC**	7-ethyl-10- [4-N-(5-aminopentanoic acid)-1-piperidino] carbonyloxy-camptothecin
**BGUS**	β-glucuronidase
**CD**	Cytidine deaminase
**CES**	Carboxylesterase
**CRC**	Colorectal cancer
**cGMP**	Current Good Manufacture Practices
**COX-2**	Cyclooxygenase-2
**CSCO**	Chinese Society of Clinical Oncology
**CYP3A4**	Cytochrome P450 isoenzyme 3A4
**DMH**	1,2-dimethylhydrazine
**EGFR**	Epidermal growth factor
**EGF**	Epidermal growth factor receptor
**ESMO**	European Society for Medical Oncology
**FDA**	Food and Drug Administration
**FOLFIRI**	Irinotecan, 5-FU/leucovorin
**FOLFOXIRI**	Irinotecan, oxaliplatin, 5-FU/leucovorin
**FdUDP**	Fluorodeoxyuridine diphosphate
**FdUMP**	Fluorodeoxyuridine monophosphate
**FdUTP**	Fluorodeoxyuridine triphosphate
**FUDP**	Fluorouridine diphosphate
**FUDR**	Fluorodeoxyuridine
**FUMP**	Fluorouridine monophosphate
**FUR**	Fluorouridine
**FUTP**	Fluorouridine triphosphate
**IARC**	International Agency for Research on Cancer
**IFN-γ**	Interferon-gamma
**iNOS**	Inducible nitric oxide synthase
**IL-6**	Interleukin-6
**IL-1β**	Interleukin-1beta
**mCRC**	Metastatic colorectal cancer
**NCCN**	National Comprehensive Cancer Network
**NF-κB**	Nuclear factor kappa-B
**NPC**	7-ethyl-10-(4-amino-1-piperidino) carbonyloxy-camptothecin
**NK**	Natural killer
**OPRT**	Orotate phosphoribosyltransferase
**OS**	Overall survival
**PFS**	Progression-free survival
**QoL**	Quality of life
**RR**	Response rate
**RNR**	Ribonucleotide reductase
**SN-38**	7-ethyl-10-hydroxy-camptothecin
**SN-38G**	SN-38 glucuronide
**TGF-α**	Transforming growth factor-alpha
**Topo I**	Topoisomerase I
**TP**	Thymidine phosphorylase
**TK**	Thymidine kinase
**TNF-α**	Tumor necrosis factor-alpha
**UGT1A1**	Uridine diphosphate-glucuronosyltransferase 1A1
**UK**	Uridine kinase
**UP**	Uridine phosphorylase
**VEGF**	Vascular endothelial growth factor
**VEGF-A**	Vascular endothelial growth factor A
**VEGFR-1**	Vascular endothelial growth factor receptor-1
**VEGFR-2**	Vascular endothelial growth factor receptor-2
**XELIRI**	Irinotecan plus capecitabine
**5-FU**	5-Fluorouracil
**5′-DFCR**	5′-deoxy-5-fluorocytidine
**5′-DFUR**	5′-deoxy-fluorouracil
